# Biodata Mobilisation of the Entomological Collections of the Schmalhausen Institute of Zoology of the National Academy of Sciences of Ukraine

**DOI:** 10.3897/BDJ.13.e152004

**Published:** 2025-05-08

**Authors:** Andriy Babytskiy, Maryna Kaliuzhna, Oleksandr Varga, Vitalii Kavurka, Alexey Prokhorov, Volodymyr Kletionkin, Kateryna Martynova, Anna Nuzhna, Svitlana Klymenko, Victor Fursov, Maksym Parkhomenko

**Affiliations:** 1 I.I. Schmalhauzen Institute of Zoology NAS of Ukraine, Kyiv, Ukraine I.I. Schmalhauzen Institute of Zoology NAS of Ukraine Kyiv Ukraine; 2 National University of Life and Environmental Sciences of Ukraine, Kyiv, Ukraine National University of Life and Environmental Sciences of Ukraine Kyiv Ukraine; 3 Dvorichna National Nature Park, Dvorichna, Kharkiv Region, Ukraine Dvorichna National Nature Park Dvorichna, Kharkiv Region Ukraine

**Keywords:** occurrence, Insecta, biodiversity, Diptera, Hymenoptera, Lepidoptera

## Abstract

**Background:**

The article represents mobilised biodata of the first part of the entomological collections of the SIZK (Schmalhausen Institute of Zoology, Kyiv) collection funds. It includes 11 occurrence datasets covering three orders and 11 families of insects: Lepidoptera (Tortricidae), Diptera (Sciaridae, Syrphidae) and Hymenoptera (Braconidae, Crabronidae, Sphecidae, Chrysididae, Ichneumonidae, Eurytomidae, Torymidae, Ormyridae). The total number of records is 12,713, based on transcription of information from the labels of pinned or slide-mounted specimens. The records cover specimens collected during 1881-2022, mostly on the territory of Ukraine, but some collections also cover the territory of other countries. The primary purpose of publishing these data is to secure the future of the SIZK collections, which are still at risk of war damage and to make the mobilised data freely and remotely accessible through GBIF.

**New information:**

In total, three orders and 11 families of insects are represented with 12,713 records: Lepidoptera (Tortricidae), Diptera (Sciaridae, Syrphidae) and Hymenoptera (Braconidae, Crabronidae, Sphecidae, Chrysididae, Ichneumonidae, Eurytomidae, Torymidae, Ormyridae).

## Introduction

According to the latest collection funds revision of the I.I. Schmalhausen Institute of Zoology NAS of Ukraine (SIZK) conducted in 2023, it contains 6,930,352 storage units, organised into 693 collections, including 626 systematic and 67 thematic (taxonomic, ecological, histological and morphological-anatomical) collections. Undoubtedly, the most valuable part of the collection is the type specimens, which include about 22,000 units (4,200 animal species), of which 1,379 are holotypes. The collection of Rivne amber comprises 4976 specimens (150 of which are types).

A collection of specimens of organisms belonging to the same family (with some exceptions) is considered to be a systematic collection in the Institute's funds.

The storage unit in the collections is both a single specimen of an organism mounted in a certain way (dry entomological specimen on a pin, permanent slide, microphotograph of an organism that is not provided for the creation of permanent slides, wet specimen in a fixative etc.) and containers with a large number of organisms (dried specimens on so-called cotton 'mattresses' (layers), containers with fixed unassembled or sorted material etc.).

The creation of institutional collections began in 1919 by the systematists of the Zoological Museum of the All-Ukrainian Academy of Sciences (the old name of the National Academy of Sciences of Ukraine) and since the establishment of the Institute of Zoology of the NAS of Ukraine in 1930. Amongst the oldest and most valuable specimens in the Fund of the Institute are the so-called memorial collections of Khristoforov, Grossheim, Lebedev, Karavaiev, Paramonov, Vainshtein, Bilanovskyi and others (Puchkov et al. 2023). Thus, zoological collections also have an important cultural and historical significance.

However, the greatest importance of the Fund collections of the Institute of Zoology is scientific, as they contain information on the biodiversity of Ukraine and other parts of the world over the last century. This is particularly important for the areas currently affected by military operations or under occupation as a result of the Russian invasion of Ukraine.

More than 70% of the collections of the Institute of Zoology are entomological. Most of the specimens were collected on the territory of modern Ukraine and the former Soviet Union, but some collections contain specimens from all over the world. Most of the collections were created in the 20^th^ century and are represented by specimens collected from the beginning of 1900 to the modern period. The aim of the first stage of digitisation of the Institute's collections is to mobilise the biodata of the largest entomological collections of the orders Hymenoptera, Diptera and Lepidoptera, which will make information on the species composition and distribution of these insects in Ukraine in the 20^th^ century available to a wide range of researchers.

## General description

### Purpose

The primary purpose of publishing these data is to secure the future of the SIZK collections, which remain at risk of damage from the war and to make the mobilised data freely and remotely accessible through GBIF. This also aims to improve the implementation of Ukrainian biodiversity data and their integrative use in international research projects.

## Project description

### Title

Biodata mobilization of entomological collections of I. I. Schmalhausen Institute of Zoology of NAS of Ukraine (Cepa-LT-2017/10049)

### Personnel

**Project PI**: Andriy Babytskiy (Dr., Senior Research Scientist, SIZK, Department of Entomology and Collection Management, ORCID https://orcid.org/0000-0003-2758-0319).

**Core Team**: Maryna Kaliuzhna (Dr., Research Scientist, SIZK, Department of Taxonomy of Entomophagous Insects and Ecological Principles of Biocontrol, ORCID https://orcid.org/0000-0002-9265-0195),

Vitalii Kavurka (Dr., Research Scientist, SIZK, Department of Entomology and Collection Management, ORCID https://orcid.org/0000-0002-2447-3588),

Alexey Prokhorov (Dr., Research Scientist, SIZK, Department of Entomology and Collection Management, ORCID https://orcid.org/0000-0002-3367-260X),

Volodymyr Kletionkin (Нead of the research department, Dvorichanskyi National Nature Park, Research Department, ORCID https://orcid.org/0000-0002-6468-3280),

Kateryna Martynova (Dr., Junior Research Scientist, SIZK, Department of Taxonomy of Entomophagous Insects and Ecological Principles of Biocontrol, ORCID https://orcid.org/0000-0002-9896-3504),

Oleksandr Varga (Dr., Senior Research Scientist, SIZK, Department of Taxonomy of Entomophagous Insects and Ecological Principles of Biocontrol, ORCID https://orcid.org/0000-0002-6285-7830),

Anna Nuzhna (Dr., Scientific Consultant, SIZK, Department of Taxonomy of Entomophagous Insects and Ecological Principles of Biocontrol, https://www.researchgate.net/profile/Anna-Nuzhna-2),

Svitlana Klymenko (Dr., Junior Research Scientist, SIZK, Department of Taxonomy of Entomophagous Insects and Ecological Principles of Biocontrol, ORCID https://orcid.org/0000-0001-8143-5070),

Victor Fursov (Dr., Senior Research Scientist, SIZK, Department of Taxonomy of Entomophagous Insects and Ecological Principles of Biocontrol, ORCID https://orcid.org/0000-0002-3318-2491),

Maksym Parkhomenko (Research Associate, Dvorichanskyi National Nature Park, Research Department, ORCID https://orcid.org/0000-0002-6366-5478).

### Study area description

In our study, we focus on the biodata mobilisation of preserved specimens from the insect collections of the I.I. Schmalhausen Institute of Zoology NAS of Ukraine. The taxonomic spectrum includes three orders and 11 families of insects: Lepidoptera (Tortricidae), Diptera (Sciaridae, Syrphidae) and Hymenoptera (Braconidae, Crabronidae, Sphecidae, Chrysididae, Ichneumonidae, Eurytomidae, Torymidae, Ormyridae). Biodata are summarised in 11 datasets and include more than 12,700 specimens.

### Funding

BioDATA grant for data mobilisation including digitisation, data quality assurance, data preparation and publication of collection specimen and other species data from Ukraine to GBIF. Data preparation was supported by the project "Biodata mobilization of entomological collections of the I. I. Schmalhausen Institute of Zoology of NAS of Ukraine" (project number Biodata Ce-pa-LT-2017/10049, UiO project number 101063).

## Sampling methods

### Sampling description

The entomological part of the SIZK funds consists of 258 systematic collections. Some of them, such as the Sciaridae, Tortricidae or largely Ichneumonidae collections, are actively formed and constantly enriched with new specimens by their curators, while the others are in archival storage. The selected collections correspond to the specialisation of our team members and results of biodata mobilisation presented in this article are only the first step towards the digitisation of the SIZK collections. The specimens from the selected collections were catalogued using Microsoft Office software. The data from the specimen labels were transcribed and translated from Ukrainian or other Slavic languages into English using the Oxford English Dictionnary, DeepL Translator, Google Translator and Slovnyk.ua for transliteration. The taxonomy was verified against the GBIF taxonomy backbone and localities were georeferenced by transferring geographical coordinates directly from labels, if available, or by searching for the specified settlement or other object on a Google map and entering the coordinates with the accuracy of determining the location of sample collection. All catalogues were transferred in separate Occurrence datasets according to the GBIF requirements.

### Quality control

Excel software was used to manually check the quality of the datasets. The datasets were then processed using OpenRefine for final data quality checks and the GBIF species matching tool for taxonomic checks.

### Step description

1. Cataloguing of the collections (transcribing all relevant information from the labels into Excel files (species names, locality, collector, date etc.). 2. Translation of primary label information from Slavic languages into English. 3. Georeferencing of localities without coordinates. 4. Preparation of occurrence datasets. 5. Data checking and cleaning. 6. Publication of datasets on GBIF.

## Geographic coverage

### Description

**Figure 1.**
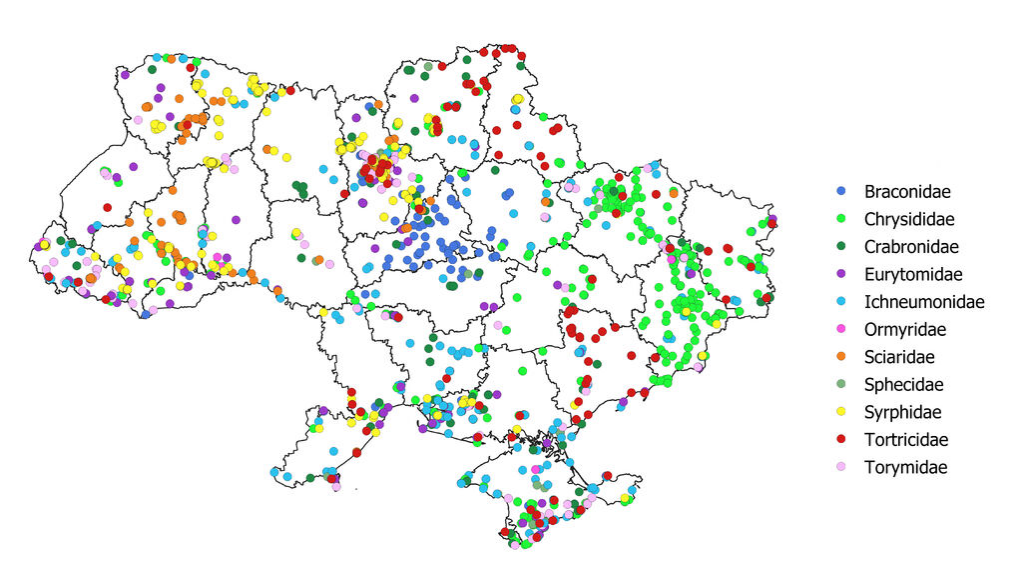
Map with species records of different insect families in Ukraine presented in this article.

**Figure 2.**
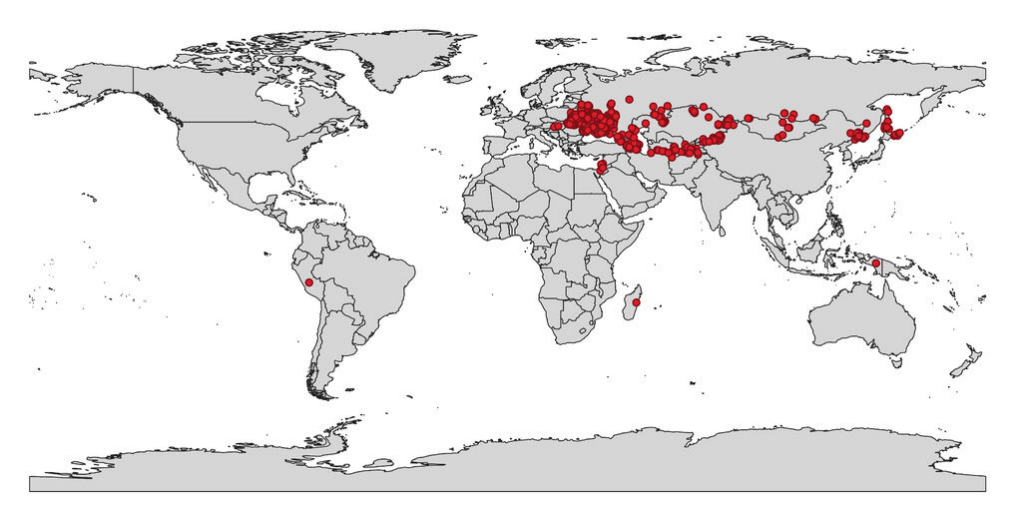
Map with insect species records in the world presented in this article.

The data mobilised in this article are mostly focused on the territory of Ukraine, with emphasis on the specimens collected in the areas suffering from the current war conflict between Ukraine and the Russian Federation (Fig. 1, Table 1). Some collections also cover the territory of other countries, namely Armenia, Azerbaijan, Belarus, China, Georgia, Hungary, Indonesia, Israel, Jordan, Kazakhstan, Kyrgyzstan, Madagascar, Moldova, Mongolia, Peru, Russian Federation, Tajikistan, Turkmenistan and Uzbekistan (Fig. 2, Suppl. material 1).

## Taxonomic coverage

### Description

The taxonomic coverage includes three orders and 11 families of insects: Lepidoptera (Tortricidae), Diptera (Sciaridae, Syrphidae) and Hymenoptera (Braconidae, Crabronidae, Sphecidae, Chrysididae, Ichneumonidae, Eurytomidae, Torymidae, Ormyridae).

## Temporal coverage

### Notes

The datasets cover specimens collected during the years 1881–2022.

## Collection data

### Collection name

I. I. Schmalhausen Institute of Zoology NAS of Ukraine

### Collection identifier

SIZK

### Specimen preservation method

Pinned and slides.

### Curatorial unit

Entomological unit.

## Usage licence

### Usage licence

Creative Commons Public Domain Waiver (CC-Zero)

## Data resources

### Data package title

Entomological unit of SIZK data mobilisation

### Number of data sets

11

### Data set 1.

#### Data set name

Sciaridae (Diptera, Sciaroidea) collection of the I.I. Schmalhausen Institute of Zoology NAS of Ukraine.

#### Data format

DarwinCore

#### Character set

UTF-8

#### Download URL

https://www.gbif.org/dataset/8703e8e3-26e8-463e-8fb3-b5275a212600

#### Description

The tab-delimited CSV-formatted dataset created following the DarwinCore standard. It contains 991 occurrence records on the specimens of sciarids deposited in the SIZK_Sciaridae collection.

**Data set 1.** 

Column label	Column description
occurrenceID	An unique identifier for the Occurrence (as opposed to a particular digital record of the occurrence).
institutionID	An identifier for the institution having custody of the object(s) or information referred to in the record i.e. http://grscicoll.org/institution/schmaulhausen-institute-zoology
collectionCode	The name, acronym, coden or initialism identifying the collection or dataset from which the record was derived.
catalogNumber	An identifier (preferably unique) for the record within the dataset or collection.
scientificName	The full scientific name, with authorship and date information of publication of the description if known.
decimalLatitude	The geographic latitude (in decimal degrees, using the spatial reference system given in geodeticDatum) of the geographic centre of a Location. Positive values are north of the Equator, negative values are south of it. Legal values lie between -90 and 90, inclusive.
decimalLongitude	The geographic longitude (in decimal degrees, using the spatial reference system given in geodeticDatum) of the geographic centre of a Location. Positive values are east of the Greenwich Meridian, negative values are west of it. Legal values lie between -180 and 180, inclusive.
minimumElevationInMeters	The lower limit of the range of elevation (altitude, usually above sea level), in metres.
stateProvince	The name of the next smaller administrative region than country (state, province, canton, department, region etc.) in which the Location occurs.
eventDate	The date-time or interval during which an Event occurred. For occurrences, this is the Year-Month-Day format (YYYY-MM-DD) when the event was recorded.
locality	The specific description of the place.
habitat	A category or description of the habitat in which the Event occurred.
occurrenceRemarks	Comments or notes about the Occurrence.
samplingProtocol	The names of, references to, or descriptions of the methods or protocols used during an Event.
sex	The sex of the biological individual(s) represented in the Occurrence.
recordedBy	A list (concatenated and separated) of names of people, groups or organisations responsible for recording the original Occurrence.
identifiedBy	A list (concatenated and separated) of names of people, groups or organisations who assigned the Taxon to the subject.
lifeStage	The age class or life stage of the Organism(s) at the time the Occurrence was recorded.
maximumElevationInMeters	The upper limit of the range of elevation (altitude, usually above sea level), in metres.
coordinateUncertaintyInMetrs	The horizontal distance (in metres) from the given decimalLatitude and decimalLongitude describing the smallest circle containing the whole of the Location.
basisOfRecord	The specific nature of the data record.
associatedTaxa	A list (concatenated and separated) of identifiers or names of taxa and the associations of this Occurrence to each of them.
taxonRank	The taxonomic rank of the most specific name in the scientificName.
kingdom	The full scientific name of the kingdom in which the taxon is classified.
phylum	The full scientific name of the phylum or division in which the taxon is classified.
class	The full scientific name of the class (i.e. Insecta) in which the taxon is classified.
order	The full scientific name of the order in which the taxon is classified.
family	The full scientific name of the family in which the taxon is classified.
genus	The full scientific name of the genus in which the taxon is classified.
subgenus	The full scientific name of the subgenus in which the taxon is classified.
country	The name of the country or major administrative unit in which the Location occurs.
geodeticDatum	The ellipsoid, geodetic datum or spatial reference system (SRS) upon which the geographic coordinates given in decimalLatitude and decimalLongitude are based.
continent	The name of the continent in which the Location occurs.
countryCode	The standard code for the country in which the Location occurs.
specificEpithet	The name of the first or species epithet of the scientificName.
license	A legal document giving official permission to do something with the resource.

### Data set 2.

#### Data set name

Syrphidae (Diptera, Brachycera) collection of the I.I. Schmalhausen Institute of Zoology NAS of Ukraine.

#### Data format

DarwinCore

#### Character set

UTF-8

#### Download URL

https://www.gbif.org/dataset/2e7346bf-be68-4dd1-8231-a8aa3801d9ad

#### Description

The tab-delimited CSV-formatted dataset created following the DarwinCore standard. It contains 2,773 occurrence records on the specimens of syrphids deposited in the SIZK_Syrphidae collection.

**Data set 2.** 

Column label	Column description
occurrenceID	An identifier for the Occurrence (as opposed to a particular digital record of the occurrence).
license	A legal document giving official permission to do something with the resource.
collectionCode	The name, acronym, coden or initialism identifying the collection or dataset from which the record was derived.
scientificName	An identifier for the nomenclatural (not taxonomic) details of a scientific name.
taxonRank	The taxonomic rank of the most specific name in the scientificName.
lifeStage	The age class or life stage of the Organism(s) at the time the Occurrence was recorded.
decimalLatitude	The geographic latitude (in decimal degrees, using the spatial reference system given in geodeticDatum) of the geographic centre of a Location. Positive values are north of the Equator, negative values are south of it. Legal values lie between -90 and 90, inclusive.
decimalLongitude	The geographic longitude (in decimal degrees, using the spatial reference system given in geodeticDatum) of the geographic centre of a Location. Positive values are east of the Greenwich Meridian, negative values are west of it. Legal values lie between -180 and 180, inclusive.
higherGeography	An identifier for the geographic region within which the Location occurred.
country	The name of the country or major administrative unit in which the Location occurs.
stateProvince	The name of the next smaller administrative region than country (state, province, canton, department, region etc.) in which the Location occurs.
locality	The specific description of the place.
eventDate	The date-time or interval during which an Event occurred. For occurrences, this is the Year-Month-Day format (YYYY-MM-DD) when the event was recorded.
habitat	A category or description of the habitat in which the Event occurred.
occurrenceRemarks	Comments or notes about the Occurrence.
samplingProtocol	The names of, references to, or descriptions of the methods or protocols used during an Event.
individualCount	The number of individuals present at the time of the Occurrence.
sex	The sex of the biological individual(s) represented in the Occurrence.
recordedBy	A list (concatenated and separated) of names of people, groups or organisations responsible for recording the original Occurrence.
identifiedBy	A list (concatenated and separated) of names of people, groups or organisations who assigned the Taxon to the subject.
basisOfRecord	The specific nature of the data record.
kingdom	The full scientific name of the kingdom in which the taxon is classified
phylum	The full scientific name of the phylum or division in which the taxon is classified.
class	The full scientific name of the class (i.e. Insecta) in which the taxon is classified.
order	The full scientific name of the order in which the taxon is classified.
family	The full scientific name of the family in which the taxon is classified.
genus	The full scientific name of the genus in which the taxon is classified.
specificEpithet	The name of the first or species epithet of the scientificName.
minimumElevationInMeters	The lower limit of the range of elevation (altitude, usually above sea level), in metres.
maximumElevationInMeters	The upper limit of the range of elevation (altitude, usually above sea level), in metres.
geodeticDatum	The ellipsoid, geodetic datum or spatial reference system (SRS) upon which the geographic coordinates given in decimalLatitude and decimalLongitude are based.
continent	The name of the continent in which the Location occurs.
countryCode	The standard code for the country in which the Location occurs.
subgenus	The full scientific name of the subgenus in which the taxon is classified.

### Data set 3.

#### Data set name

Tortricidae (Lepidoptera) collection of the I.I. Schmalhausen Institute of Zoology NAS of Ukraine.

#### Data format

DarwinCore

#### Character set

UTF-8

#### Download URL

https://www.gbif.org/dataset/c568eff5-02c9-4a05-9e01-b46ec0c0f3d5

#### Description

The tab-delimited CSV-formatted dataset created following the DarwinCore standard. It contains 2,005 occurrence records on the specimens of Tortricidae family deposited in the SIZK_Tortricidae collection.

**Data set 3.** 

Column label	Column description
occurrenceID	An identifier for the Occurrence (as opposed to a particular digital record of the occurrence).
collectionCode	The name, acronym, coden or initialism identifying the collection or dataset from which the record was derived.
scientificName	The full scientific name, with authorship and date information of publication of the description if known.
taxonRank	The taxonomic rank of the most specific name in the scientificName.
lifeStage	The age class or life stage of the Organism(s) at the time the Occurrence was recorded.
decimalLatitude	The geographic latitude (in decimal degrees, using the spatial reference system given in geodeticDatum) of the geographic centre of a Location. Positive values are north of the Equator, negative values are south of it. Legal values lie between -90 and 90, inclusive.
decimalLongitude	The geographic longitude (in decimal degrees, using the spatial reference system given in geodeticDatum) of the geographic centre of a Location. Positive values are east of the Greenwich Meridian, negative values are west of it. Legal values lie between -180 and 180, inclusive.
minimumElevationInMeters	The lower limit of the range of elevation (altitude, usually above sea level), in metres.
maximumElevationInMeters	The upper limit of the range of elevation (altitude, usually above sea level), in metres.
higherGeography	A list (concatenated and separated) of geographic names less specific than the information captured in the locality term.
country	The name of the country or major administrative unit in which the Location occurs.
stateProvince	The name of the next smaller administrative region than country (state, province, canton, department, region etc.) in which the Location occurs.
locality	The specific description of the place.
eventDate	The date-time or interval during which an Event occurred. For occurrences, this is the Year-Month-Day format (YYYY-MM-DD) when the event was recorded.
habitat	A category or description of the habitat in which the Event occurred.
occurrenceRemarks	Comments or notes about the Occurrence.
samplingProtocol	The names of, references to, or descriptions of the methods or protocols used during an Event.
individualCount	The number of individuals present at the time of the Occurrence.
sex	The sex of the biological individual(s) represented in the Occurrence.
recordedBy	A list (concatenated and separated) of names of people, groups or organisations responsible for recording the original Occurrence.
identifiedBy	A list (concatenated and separated) of names of people, groups or organisations who assigned the Taxon to the subject.
basisOfRecord	The specific nature of the data record.
kingdom	The full scientific name of the kingdom in which the taxon is classified.
phylum	The full scientific name of the phylum or division in which the taxon is classified.
class	The full scientific name of the class (i.e. Insecta) in which the taxon is classified.
order	The full scientific name of the order in which the taxon is classified.
family	The full scientific name of the family in which the taxon is classified.
genus	The full scientific name of the genus in which the taxon is classified.
specificEpithet	The name of the first or species epithet of the scientificName.
geodeticDatum	The ellipsoid, geodetic datum or spatial reference system (SRS) upon which the geographic coordinates given in decimalLatitude and decimalLongitude are based.
continent	The name of the continent in which the Location occurs.
license	A legal document giving official permission to do something with the resource.

### Data set 4.

#### Data set name

Chrysididae (Hymenoptera, Apocrita) collection of the I.I. Schmalhausen Institute of Zoology NAS of Ukraine.

#### Data format

DarwinCore

#### Character set

UTF-8

#### Download URL

https://www.gbif.org/dataset/d3e53ab3-8946-4ad9-b481-b75ff6179057

#### Description

The tab-delimited CSV-formatted dataset created following the DarwinCore standard. It contains 2,489 occurrence records on the specimens of chrysidid wasps deposited in the SIZK_Chrysididae collection.

**Data set 4.** 

Column label	Column description
occurrenceID	An identifier for the Occurrence (as opposed to a particular digital record of the occurrence).
basisOfRecord	The specific nature of the data record.
eventDate	The date-time or interval during which an Event occurred. For occurrences, this is the Year-Month-Day format (YYYY-MM-DD) when the event was recorded.
year	The four-digit year in which the Event occurred, according to the Common Era Calendar.
month	The integer month in which the Event occurred.
day	The integer day of the month on which the Event occurred.
institutionCode	The name (or acronym) in use by the institution having custody of the object(s) or information referred to in the record.
eventRemarks	Comments or notes about the Event.
scientificName	The full scientific name, with authorship and date information of publication of the description if known.
kingdom	The full scientific name of the kingdom in which the taxon is classified.
phylum	The full scientific name of the phylum or division in which the taxon is classified.
class	The full scientific name of the class (i.e. Insecta) in which the taxon is classified.
order	The full scientific name of the order in which the taxon is classified.
family	The full scientific name of the family in which the taxon is classified.
genus	The full scientific name of the genus in which the taxon is classified.
specificEpithet	The name of the first or species epithet of the scientificName.
infraspecificEpithet	The name of the lowest or terminal infraspecific epithet of the scientificName, excluding any rank designation.
taxonRank	The taxonomic rank of the most specific name in the scientificName.
identifiedBy	A list (concatenated and separated) of names of people, groups or organisations who assigned the Taxon to the subject.
continent	The name of the continent in which the Location occurs.
country	The name of the country or major administrative unit in which the Location occurs.
countryCode	The standard code for the country in which the Location occurs.
stateProvince	The name of the next smaller administrative region than country (state, province, canton, department, region etc.) in which the Location occurs.
county	The full, unabbreviated name of the next smaller administrative region than stateProvince (county, shire, department etc.) in which the Location occurs.
locality	The specific description of the place.
verbatimLocality	The original textual description of the place.
locationAccordingTo	Information about the source of this Location information. Could be a publication (gazetteer), institution or team of individuals.
decimalLatitude	The geographic latitude (in decimal degrees, using the spatial reference system given in geodeticDatum) of the geographic centre of a Location. Positive values are north of the Equator, negative values are south of it. Legal values lie between -90 and 90, inclusive.
decimalLongitude	The geographic longitude (in decimal degrees, using the spatial reference system given in geodeticDatum) of the geographic centre of a Location. Positive values are east of the Greenwich Meridian, negative values are west of it. Legal values lie between -180 and 180, inclusive.
geodeticDatum	The ellipsoid, geodetic datum or spatial reference system (SRS) upon which the geographic coordinates given in decimalLatitude and decimalLongitude are based.
georeferencedBy	A list (concatenated and separated) of names of people, groups or organisations who determined the georeference (spatial representation) for the Location.
georeferenceProtocol	A description or reference to the methods used to determine the spatial footprint, coordinates and uncertainties.
collectionCode	The name, acronym, coden or initialism identifying the collection or dataset from which the record was derived.
organismQuantity	A number or enumeration value for the quantity of organisms.
organismQuantityType	The type of quantification system used for the quantity of organisms.
sex	The sex of the biological individual(s) represented in the Occurrence.
lifeStage	The age class or life stage of the Organism(s) at the time the Occurrence was recorded.
associatedTaxa	A list (concatenated and separated) of identifiers or names of taxa and the associations of this Occurrence to each of them.
type	The nature or genre of the resource.
language	The language of the resource i.e. English.
license	A legal document giving official permission to do something with the resource.

### Data set 5.

#### Data set name

Tribes Larrini, Miscophini (Hymenoptera, Crabronidae) in collection of the I.I. Schmalhausen Institute of Zoology NAS of Ukraine.

#### Data format

DarwinCore

#### Character set

UTF-8

#### Download URL

https://www.gbif.org/dataset/8ec33d8f-5b58-4dc7-b71d-4e32d3ae99e7

#### Description

The tab-delimited CSV-formatted dataset created following the DarwinCore standard. It contains 250 occurrence records on the specimens of Larrini and Miscophini tribes of Crabronidae family. Material deposited in the SIZK_Crabronidae collection.

**Data set 5.** 

Column label	Column description
occurrenceID	An identifier for the Occurrence (as opposed to a particular digital record of the occurrence).
institutionID	An identifier for the institution having custody of the object(s) or information referred to in the record i.e. http://grscicoll.org/institution/schmaulhausen-institute-zoology
collectionCode	The name, acronym, coden or initialism identifying the collection or dataset from which the record was derived.
basisOfRecord	The specific nature of the data record.
eventDate	The date-time or interval during which an Event occurred. For occurrences, this is the Year-Month-Day format (YYYY-MM-DD) when the event was recorded.
year	The four-digit year in which the Event occurred, according to the Common Era Calendar.
month	The integer month in which the Event occurred.
day	The integer day of the month on which the Event occurred.
verbatimEventDate	The verbatim original representation of the date and time information for an Event.
eventRemarks	Comments or notes about the Event.
scientificName	An identifier for the nomenclatural (not taxonomic) details of a scientific name.
kingdom	The full scientific name of the kingdom in which the taxon is classified.
phylum	The full scientific name of the phylum or division in which the taxon is classified.
class	The full scientific name of the class (i.e. Insecta) in which the taxon is classified.
order	The full scientific name of the order in which the taxon is classified.
family	The full scientific name of the family in which the taxon is classified.
genus	The full scientific name of the genus in which the taxon is classified.
specificEpithet	The name of the first or species epithet of the scientificName.
infraspecificEpithet	The name of the lowest or terminal infraspecific epithet of the scientificName, excluding any rank designation.
taxonRank	The taxonomic rank of the most specific name in the scientificName.
identifiedBy	A list (concatenated and separated) of names of people, groups or organisations who assigned the Taxon to the subject.
dateIdentified	The date on which the subject was determined as representing the Taxon.
continent	The name of the continent in which the Location occurs.
country	The name of the country or major administrative unit in which the Location occurs.
countryCode	The standard code for the country in which the Location occurs.
stateProvince	The name of the next smaller administrative region than country (state, province, canton, department, region etc.) in which the Location occurs.
county	The full, unabbreviated name of the next smaller administrative region than stateProvince (county, shire, department etc.) in which the Location occurs.
municipality	The full, unabbreviated name of the next smaller administrative region than county (city, municipality etc.) in which the Location occurs.
locality	The specific description of the place.
verbatimLocality	The original textual description of the place.
habitat	A category or description of the habitat in which the Event occurred.
decimalLatitude	The geographic latitude (in decimal degrees, using the spatial reference system given in geodeticDatum) of the geographic centre of a Location. Positive values are north of the Equator, negative values are south of it. Legal values lie between -90 and 90, inclusive.
decimalLongitude	The geographic longitude (in decimal degrees, using the spatial reference system given in geodeticDatum) of the geographic centre of a Location. Positive values are east of the Greenwich Meridian, negative values are west of it. Legal values lie between -180 and 180, inclusive.
geodeticDatum	The ellipsoid, geodetic datum or spatial reference system (SRS) upon which the geographic coordinates given in decimalLatitude and decimalLongitude are based.
coordinateUncertaintyInMeters	The horizontal distance (in metres) from the given decimalLatitude and decimalLongitude describing the smallest circle containing the whole of the Location.
georeferencedBy	A list (concatenated and separated) of names of people, groups or organisations who determined the georeference (spatial representation) for the Location.
georeferencedDate	The date on which the Location was georeferenced.
georeferenceProtocol	A description or reference to the methods used to determine the spatial footprint, coordinates and uncertainties.
minimumElevationInMeters	The lower limit of the range of elevation (altitude, usually above sea level), in metres.
organismQuantity	A number or enumeration value for the quantity of organisms.
organismQuantityType	The type of quantification system used for the quantity of organisms.
sex	The sex of the biological individual(s) represented in the Occurrence.
lifeStage	The age class or life stage of the Organism(s) at the time the Occurrence was recorded.
establishmentMeans	Statement about whether an organism or organisms have been introduced to a given place and time through the direct or indirect activity of modern humans.
type	The nature or genre of the resource.
language	The language of the resource i.e. English.
license	A legal document giving official permission to do something with the resource.
institutionCode	The name (or acronym) in use by the institution having custody of the object(s) or information referred to in the record.
catalogNumber	An identifier (preferably unique) for the record within the dataset or collection.
maximumElevationInMeters	The upper limit of the range of elevation (altitude, usually above sea level), in metres.

### Data set 6.

#### Data set name

Crabronidae, Sphecidae (Hymenoptera) collection of the I.I. Schmalhausen Institute of Zoology NAS of Ukraine.

#### Data format

DarwinCore

#### Character set

UTF-8

#### Download URL

https://www.gbif.org/dataset/407b1d4c-71d8-4d7f-84c3-4839b022845c

#### Description

The tab-delimited CSV-formatted dataset created following the DarwinCore standard. It contains 1,070 occurrence records on the specimens of Crabronidae and Sphecidae families. Material deposited in the SIZK_Crabronidae and SIZK_Sphecidae collections.

**Data set 6.** 

Column label	Column description
occurrenceID	An identifier for the Occurrence (as opposed to a particular digital record of the occurrence).
institutionID	An identifier for the institution having custody of the object(s) or information referred to in the record i.e. http://grscicoll.org/institution/schmaulhausen-institute-zoology
collectionCode	The name, acronym, coden or initialism identifying the collection or dataset from which the record was derived.
catalogNumber	An identifier (preferably unique) for the record within the dataset or collection.
scientificName	An identifier for the nomenclatural (not taxonomic) details of a scientific name.
decimalLatitude	The geographic latitude (in decimal degrees, using the spatial reference system given in geodeticDatum) of the geographic centre of a Location. Positive values are north of the Equator, negative values are south of it. Legal values lie between -90 and 90, inclusive.
decimalLongitude	The geographic longitude (in decimal degrees, using the spatial reference system given in geodeticDatum) of the geographic centre of a Location. Positive values are east of the Greenwich Meridian, negative values are west of it. Legal values lie between -180 and 180, inclusive.
coordinateUncertaintyInMeters	The horizontal distance (in metres) from the given decimalLatitude and decimalLongitude describing the smallest circle containing the whole of the Location.
minimumElevationInMeters	The lower limit of the range of elevation (altitude, usually above sea level), in metres.
maximumElevationInMeters	The upper limit of the range of elevation (altitude, usually above sea level), in metres.
continent	The name of the continent in which the Location occurs.
country	The name of the country or major administrative unit in which the Location occurs.
countryCode	The standard code for the country in which the Location occurs.
stateProvince	The name of the next smaller administrative region than country (state, province, canton, department, region etc.) in which the Location occurs.
eventDate	The date-time or interval during which an Event occurred. For occurrences, this is the Year-Month-Day format (YYYY-MM-DD) when the event was recorded.
locality	The specific description of the place.
habitat	A category or description of the habitat in which the Event occurred.
geodeticDatum	The ellipsoid, geodetic datum or spatial reference system (SRS) upon which the geographic coordinates given in decimalLatitude and decimalLongitude are based.
sex	The sex of the biological individual(s) represented in the Occurrence.
recordedBy	A list (concatenated and separated) of names of people, groups or organisations responsible for recording the original Occurrence.
georeferencedBy	A list (concatenated and separated) of names of people, groups or organisations who determined the georeference (spatial representation) for the Location.
georeferencedDate	The date on which the Location was georeferenced.
georeferenceProtocol	A description or reference to the methods used to determine the spatial footprint, coordinates and uncertainties.
identifiedBy	A list (concatenated and separated) of names of people, groups or organisations who assigned the Taxon to the subject.
dateIdentified	The date on which the subject was determined as representing the Taxon.
basisOfRecord	The specific nature of the data record.
kingdom	The full scientific name of the kingdom in which the taxon is classified.
phylum	The full scientific name of the phylum or division in which the taxon is classified.
class	The full scientific name of the class (i.e. Insecta) in which the taxon is classified.
order	The full scientific name of the order in which the taxon is classified.
family	The full scientific name of the family in which the taxon is classified.
genus	The full scientific name of the genus in which the taxon is classified.
specificEpithet	The name of the first or species epithet of the scientificName.
infraspecificEpithet	The name of the lowest or terminal infraspecific epithet of the scientificName, excluding any rank designation.
occurrenceRemarks	Comments or notes about the Occurrence.
verbatimLocality	The original textual description of the place.
taxonRank	The taxonomic rank of the most specific name in the scientificName.
license	A legal document giving official permission to do something with the resource.

### Data set 7.

#### Data set name

Eurytomidae, Torymidae, Ormyridae (Hymenoptera, Apocrita) collection of the I.I. Schmalhausen Institute of Zoology NAS of Ukraine

#### Data format

DarwinCore

#### Character set

UTF-8

#### Download URL

https://www.gbif.org/dataset/f9531927-8a71-4807-b484-e5f9d472d1a2

#### Description

The tab-delimited CSV-formatted dataset created following the DarwinCore standard. It contains 465 occurrence records on the specimens of Eurytomidae, Torymidae and Ormyridae families. Material deposited in the SIZK_Eurytomidae, SIZK_Torymidae and SIZK_Ormyridae collections.

**Data set 7.** 

Column label	Column description
occurrenceID	An identifier for the Occurrence (as opposed to a particular digital record of the occurrence).
basisOfRecord	The specific nature of the data record.
eventDate	The date-time or interval during which an Event occurred. For occurrences, this is the Year-Month-Day format (YYYY-MM-DD) when the event was recorded.
year	The four-digit year in which the Event occurred, according to the Common Era Calendar.
month	The integer month in which the Event occurred.
day	The integer day of the month on which the Event occurred.
verbatimEventDate	The verbatim original representation of the date and time information for an Event.
scientificName	An identifier for the nomenclatural (not taxonomic) details of a scientific name.
kingdom	The full scientific name of the kingdom in which the taxon is classified.
phylum	The full scientific name of the phylum or division in which the taxon is classified.
class	The full scientific name of the class (i.e. Insecta) in which the taxon is classified.
order	The full scientific name of the order in which the taxon is classified.
family	The full scientific name of the family in which the taxon is classified.
genus	The full scientific name of the genus in which the taxon is classified.
specificEpithet	The name of the first or species epithet of the scientificName.
taxonRank	The taxonomic rank of the most specific name in the scientificName.
identifiedBy	A list (concatenated and separated) of names of people, groups or organisations who assigned the Taxon to the subject.
continent	The name of the continent in which the Location occurs.
country	The name of the country or major administrative unit in which the Location occurs.
countryCode	The standard code for the country in which the Location occurs.
stateProvince	The name of the next smaller administrative region than country (state, province, canton, department, region etc.) in which the Location occurs.
county	The full, unabbreviated name of the next smaller administrative region than stateProvince (county, shire, department etc.) in which the Location occurs.
municipality	The full, unabbreviated name of the next smaller administrative region than county (city, municipality etc.) in which the Location occurs.
locality	The specific description of the place.
verbatimLocality	The original textual description of the place.
habitat	A category or description of the habitat in which the Event occurred.
decimalLatitude	The geographic latitude (in decimal degrees, using the spatial reference system given in geodeticDatum) of the geographic centre of a Location. Positive values are north of the Equator, negative values are south of it. Legal values lie between -90 and 90, inclusive.
decimalLongitude	The geographic longitude (in decimal degrees, using the spatial reference system given in geodeticDatum) of the geographic centre of a Location. Positive values are east of the Greenwich Meridian, negative values are west of it. Legal values lie between -180 and 180, inclusive.
geodeticDatum	The ellipsoid, geodetic datum, or spatial reference system (SRS) upon which the geographic coordinates given in decimalLatitude and decimalLongitude are based.
coordinateUncertaintyInMeters	The horizontal distance (in metres) from the given decimalLatitude and decimalLongitude describing the smallest circle containing the whole of the Location.
license	A legal document giving official permission to do something with the resource.
georeferencedBy	A list (concatenated and separated) of names of people, groups or organisations who determined the georeference (spatial representation) for the Location.
georeferencedDate	The date on which the Location was georeferenced.
georeferenceProtocol	A description or reference to the methods used to determine the spatial footprint, coordinates and uncertainties.
georeferenceVerificationStatus	A categorical description of the extent to which the georeference has been verified to represent the best possible spatial description for the Location of the Occurrence.
associatedReferences	A list (concatenated and separated) of identifiers (publication, bibliographic reference, global unique identifier, URI) of literature associated with the Occurrence.
organismQuantity	A number or enumeration value for the quantity of organisms.
organismQuantityType	The type of quantification system used for the quantity of organisms.
sex	The sex of the biological individual(s) represented in the Occurrence.
lifeStage	The age class or life stage of the Organism(s) at the time the Occurrence was recorded.
associatedTaxa	A list (concatenated and separated) of identifiers or names of taxa and the associations of this Occurrence to each of them.
establishmentMeans	Statement about whether an organism or organisms have been introduced to a given place and time through the direct or indirect activity of modern humans.
type	The nature or genre of the resource.
language	The language of the resource i.e. English.
institutionCode	The name (or acronym) in use by the institution having custody of the object(s) or information referred to in the record.
collectionCode	The name, acronym, coden or initialism identifying the collection or dataset from which the record was derived.
previousIdentifications	A list (concatenated and separated) of previous assignments of names to the Organism.
recordedBy	A list (concatenated and separated) of names of people, groups or organisations responsible for recording the original Occurrence.

### Data set 8.

#### Data set name

Ichneumonidae (Hymenoptera, Apocrita) collection of the I.I. Schmalhausen Institute of Zoology NAS of Ukraine.

#### Data format

DarwinCore

#### Character set

UTF-8

#### Download URL

https://www.gbif.org/dataset/5b189ba4-752e-4484-bd84-28c375c4cfb2

#### Description

The tab-delimited CSV-formatted dataset created following the DarwinCore standard. It contains 1,000 occurrence records on the specimens of Ichneumonidae family. Material deposited in the SIZK_Ichneumonidae collection.

**Data set 8.** 

Column label	Column description
occurrenceID	An identifier for the Occurrence (as opposed to a particular digital record of the occurrence).
basisOfRecord	The specific nature of the data record.
eventDate	The date-time or interval during which an Event occurred. For occurrences, this is the Year-Month-Day format (YYYY-MM-DD) when the event was recorded.
year	The four-digit year in which the Event occurred, according to the Common Era Calendar.
month	The integer month in which the Event occurred.
day	The integer day of the month on which the Event occurred.
verbatimEventDate	The verbatim original representation of the date and time information for an Event.
eventRemarks	Comments or notes about the Event.
scientificName	An identifier for the nomenclatural (not taxonomic) details of a scientific name.
kingdom	The full scientific name of the kingdom in which the taxon is classified.
phylum	The full scientific name of the phylum or division in which the taxon is classified.
class	The full scientific name of the class (i.e. Insecta) in which the taxon is classified.
order	The full scientific name of the order in which the taxon is classified.
family	The full scientific name of the family in which the taxon is classified.
genus	The full scientific name of the genus in which the taxon is classified.
specificEpithet	The name of the first or species epithet of the scientificName.
taxonRank	The taxonomic rank of the most specific name in the scientificName.
identifiedBy	A list (concatenated and separated) of names of people, groups or organisations who assigned the Taxon to the subject.
recordedBy	A list (concatenated and separated) of names of people, groups or organisations responsible for recording the original Occurrence.
continent	The name of the continent in which the Location occurs.
country	The name of the country or major administrative unit in which the Location occurs.
countryCode	The standard code for the country in which the Location occurs.
stateProvince	The name of the next smaller administrative region than country (state, province, canton, department, region etc.) in which the Location occurs.
county	The full, unabbreviated name of the next smaller administrative region than stateProvince (county, shire, department etc.) in which the Location occurs.
municipality	The full, unabbreviated name of the next smaller administrative region than county (city, municipality etc.) in which the Location occurs.
locality	The specific description of the place.
verbatimLocality	The original textual description of the place.
habitat	A category or description of the habitat in which the Event occurred.
decimalLatitude	The geographic latitude (in decimal degrees, using the spatial reference system given in geodeticDatum) of the geographic centre of a Location. Positive values are north of the Equator, negative values are south of it. Legal values lie between -90 and 90, inclusive.
decimalLongitude	The geographic longitude (in decimal degrees, using the spatial reference system given in geodeticDatum) of the geographic centre of a Location. Positive values are east of the Greenwich Meridian, negative values are west of it. Legal values lie between -180 and 180, inclusive.
geodeticDatum	The ellipsoid, geodetic datum or spatial reference system (SRS) upon which the geographic coordinates given in decimalLatitude and decimalLongitude are based.
georeferencedBy	A list (concatenated and separated) of names of people, groups or organisations who determined the georeference (spatial representation) for the Location.
georeferencedDate	The date on which the Location was georeferenced.
georeferenceProtocol	A description or reference to the methods used to determine the spatial footprint, coordinates and uncertainties.
georeferenceVerificationStatus	A categorical description of the extent to which the georeference has been verified to represent the best possible spatial description for the Location of the Occurrence.
associatedReferences	A list (concatenated and separated) of identifiers (publication, bibliographic reference, global unique identifier, URI) of literature associated with the Occurrence.
organismQuantity	A number or enumeration value for the quantity of organisms.
organismQuantityType	The type of quantification system used for the quantity of organisms.
sex	The sex of the biological individual(s) represented in the Occurrence.
lifeStage	The age class or life stage of the Organism(s) at the time the Occurrence was recorded.
establishmentMeans	Statement about whether an organism or organisms have been introduced to a given place and time through the direct or indirect activity of modern humans.
type	The nature or genre of the resource.
language	The language of the resource i.e. English.
license	A legal document giving official permission to do something with the resource.
institutionCode	The name (or acronym) in use by the institution having custody of the object(s) or information referred to in the record.
collectionCode	The name, acronym, coden or initialism identifying the collection or dataset from which the record was derived.

### Data set 9.

#### Data set name

Anomaloninae (Hymenoptera, Ichneumonidae) collection of the I.I. Schmalhausen Institute of Zoology NAS of Ukraine.

#### Data format

DarwinCore

#### Character set

UTF-8

#### Download URL

https://www.gbif.org/dataset/a54fd845-5a85-4988-8f85-21731899a7a7

#### Description

The tab-delimited CSV-formatted dataset created following the DarwinCore standard. It contains 704 occurrence records on the specimens of Anomaloninae subfamily of Ichneumonidae. Material deposited in the SIZK_Ichneumonidae collection.

**Data set 9.** 

Column label	Column description
occurrenceID	An identifier for the Occurrence (as opposed to a particular digital record of the occurrence).
basisOfRecord	The specific nature of the data record.
eventDate	The date-time or interval during which an Event occurred. For occurrences, this is the Year-Month-Day format (YYYY-MM-DD) when the event was recorded.
year	The four-digit year in which the Event occurred, according to the Common Era Calendar.
month	The integer month in which the Event occurred.
day	The integer day of the month on which the Event occurred.
verbatimEventDate	The verbatim original representation of the date and time information for an Event.
eventRemarks	Comments or notes about the Event.
typeStatus	A list (concatenated and separated) of nomenclatural types (type status, typified scientific name, publication) applied to the subject.
scientificName	An identifier for the nomenclatural (not taxonomic) details of a scientific name.
kingdom	The full scientific name of the kingdom in which the taxon is classified.
phylum	The full scientific name of the phylum or division in which the taxon is classified.
class	The full scientific name of the class (i.e. Insecta) in which the taxon is classified.
order	The full scientific name of the order in which the taxon is classified.
family	The full scientific name of the family in which the taxon is classified.
genus	The full scientific name of the genus in which the taxon is classified.
specificEpithet	The name of the first or species epithet of the scientificName.
taxonRank	The taxonomic rank of the most specific name in the scientificName.
identifiedBy	A list (concatenated and separated) of names of people, groups or organisations who assigned the Taxon to the subject.
dateIdentified	The date on which the subject was determined as representing the Taxon.
continent	The name of the continent in which the Location occurs.
country	The name of the country or major administrative unit in which the Location occurs.
countryCode	The standard code for the country in which the Location occurs.
stateProvince	The name of the next smaller administrative region than country (state, province, canton, department, region etc.) in which the Location occurs.
county	The full, unabbreviated name of the next smaller administrative region than stateProvince (county, shire, department etc.) in which the Location occurs.
municipality	The full, unabbreviated name of the next smaller administrative region than county (city, municipality etc.) in which the Location occurs.
locality	The specific description of the place.
habitat	A category or description of the habitat in which the Event occurred.
decimalLatitude	The geographic latitude (in decimal degrees, using the spatial reference system given in geodeticDatum) of the geographic centre of a Location. Positive values are north of the Equator, negative values are south of it. Legal values lie between -90 and 90, inclusive.
decimalLongitude	The geographic longitude (in decimal degrees, using the spatial reference system given in geodeticDatum) of the geographic centre of a Location. Positive values are east of the Greenwich Meridian, negative values are west of it. Legal values lie between -180 and 180, inclusive.
geodeticDatum	The ellipsoid, geodetic datum or spatial reference system (SRS) upon which the geographic coordinates given in decimalLatitude and decimalLongitude are based.
coordinateUncertaintyInMeters	The horizontal distance (in metres) from the given decimalLatitude and decimalLongitude describing the smallest circle containing the whole of the Location.
verbatimCoordinates	The verbatim original spatial coordinates of the Location. The coordinate ellipsoid, geodeticDatum or full Spatial Reference System (SRS) for these coordinates should be stored in verbatimSRS and the coordinate system should be stored in verbatimCoordinateSystem.
verbatimCoordinateSystem	The coordinate format for the verbatimLatitude and verbatimLongitude or the verbatimCoordinates of the Location.
georeferencedBy	A list (concatenated and separated) of names of people, groups or organisations who determined the georeference (spatial representation) for the Location.
georeferencedDate	The date on which the Location was georeferenced.
georeferenceProtocol	A description or reference to the methods used to determine the spatial footprint, coordinates and uncertainties.
georeferenceVerificationStatus	A categorical description of the extent to which the georeference has been verified to represent the best possible spatial description for the Location of the Occurrence.
verbatimLocality	The original textual description of the place.
organismQuantity	A number or enumeration value for the quantity of organisms.
organismQuantityType	The type of quantification system used for the quantity of organisms.
sex	The sex of the biological individual(s) represented in the Occurrence.
lifeStage	The age class or life stage of the Organism(s) at the time the Occurrence was recorded.
associatedTaxa	A list (concatenated and separated) of identifiers or names of taxa and the associations of this Occurrence to each of them.
establishmentMeans	Statement about whether an organism or organisms have been introduced to a given place and time through the direct or indirect activity of modern humans.
type	The nature or genre of the resource.
language	The language of the resource i.e. English.
license	A legal document giving official permission to do something with the resource.
institutionCode	The name (or acronym) in use by the institution having custody of the object(s) or information referred to in the record.
collectionCode	The name, acronym, coden or initialism identifying the collection or dataset from which the record was derived.
occurrenceRemarks	Comments or notes about the Occurrence.
recordedBy	A list (concatenated and separated) of names of people, groups or organisations responsible for recording the original Occurrence.
minimumElevationInMeters	The lower limit of the range of elevation (altitude, usually above sea level), in metres.
maximumElevationInMeters	The upper limit of the range of elevation (altitude, usually above sea level), in metres.

### Data set 10.

#### Data set name

Ophioninae (Hymenoptera, Ichneumonidae) collection of the I.I. Schmalhausen Institute of Zoology NAS of Ukraine.

#### Data format

DarwinCore

#### Character set

UTF-8

#### Download URL

https://www.gbif.org/dataset/617835c4-d0e2-40c4-9898-9aa0b29d8108

#### Description

The tab-delimited CSV-formatted dataset created following the DarwinCore standard. It contains 517 occurrence records on the specimens of Ophioninae subfamily of Ichneumonidae. Material deposited in the SIZK_Ichneumonidae collection.

**Data set 10.** 

Column label	Column description
occurrenceID	An identifier for the Occurrence (as opposed to a particular digital record of the occurrence).
basisOfRecord	The specific nature of the data record.
eventDate	The date-time or interval during which an Event occurred. For occurrences, this is the Year-Month-Day format (YYYY-MM-DD) when the event was recorded.
year	The four-digit year in which the Event occurred, according to the Common Era Calendar.
month	The integer month in which the Event occurred.
day	The integer day of the month on which the Event occurred.
verbatimEventDate	The verbatim original representation of the date and time information for an Event.
eventRemarks	Comments or notes about the Event.
scientificName	An identifier for the nomenclatural (not taxonomic) details of a scientific name.
kingdom	The full scientific name of the kingdom in which the taxon is classified.
phylum	The full scientific name of the phylum or division in which the taxon is classified.
class	The full scientific name of the class (i.e. Insecta) in which the taxon is classified.
order	The full scientific name of the order in which the taxon is classified.
family	The full scientific name of the family in which the taxon is classified.
genus	The full scientific name of the genus in which the taxon is classified.
specificEpithet	The name of the first or species epithet of the scientificName.
taxonRank	The taxonomic rank of the most specific name in the scientificName.
identifiedBy	A list (concatenated and separated) of names of people, groups or organisations who assigned the Taxon to the subject.
minimumElevationInMeters	The lower limit of the range of elevation (altitude, usually above sea level), in metres.
continent	The name of the continent in which the Location occurs.
country	The name of the country or major administrative unit in which the Location occurs.
countryCode	The standard code for the country in which the Location occurs.
stateProvince	The name of the next smaller administrative region than country (state, province, canton, department, region etc.) in which the Location occurs.
county	The full, unabbreviated name of the next smaller administrative region than stateProvince (county, shire, department etc.) in which the Location occurs.
municipality	The full, unabbreviated name of the next smaller administrative region than county (city, municipality etc.) in which the Location occurs.
locality	The specific description of the place.
verbatimLocality	The original textual description of the place.
habitat	A category or description of the habitat in which the Event occurred.
decimalLatitude	The geographic latitude (in decimal degrees, using the spatial reference system given in geodeticDatum) of the geographic centre of a Location. Positive values are north of the Equator, negative values are south of it. Legal values lie between -90 and 90, inclusive.
decimalLongitude	The geographic longitude (in decimal degrees, using the spatial reference system given in geodeticDatum) of the geographic centre of a Location. Positive values are east of the Greenwich Meridian, negative values are west of it. Legal values lie between -180 and 180, inclusive.
geodeticDatum	The ellipsoid, geodetic datum or spatial reference system (SRS) upon which the geographic coordinates given in decimalLatitude and decimalLongitude are based.
coordinateUncertaintyInMeters	The horizontal distance (in metres) from the given decimalLatitude and decimalLongitude describing the smallest circle containing the whole of the Location.
georeferencedBy	A list (concatenated and separated) of names of people, groups or organisations who determined the georeference (spatial representation) for the Location.
georeferencedDate	The date on which the Location was georeferenced.
georeferenceProtocol	A description or reference to the methods used to determine the spatial footprint, coordinates and uncertainties.
maximumElevationInMeters	The upper limit of the range of elevation (altitude, usually above sea level), in metres.
organismQuantity	A number or enumeration value for the quantity of organisms.
organismQuantityType	The type of quantification system used for the quantity of organisms.
sex	The sex of the biological individual(s) represented in the Occurrence.
lifeStage	The age class or life stage of the Organism(s) at the time the Occurrence was recorded.
establishmentMeans	Statement about whether an organism or organisms have been introduced to a given place and time through the direct or indirect activity of modern humans.
type	The nature or genre of the resource.
language	The language of the resource i.e. English.
license	A legal document giving official permission to do something with the resource.
institutionCode	The name (or acronym) in use by the institution having custody of the object(s) or information referred to in the record.
collectionCode	The name, acronym, coden or initialism identifying the collection or dataset from which the record was derived.

### Data set 11.

#### Data set name

Aphidiinae (Hymenoptera, Braconidae) collection of the I.I. Schmalhausen Institute of Zoology NAS of Ukraine.

#### Data format

DarwinCore

#### Character set

UTF-8

#### Download URL

https://www.gbif.org/dataset/3b153a6b-491c-4f58-bc85-34c2853efed3

#### Description

The tab-delimited CSV-formatted dataset created following the DarwinCore standard. It contains 449 occurrence records on the specimens of Aphidiinae braconids deposited in the SIZK_Braconidae collection.

**Data set 11.** 

Column label	Column description
occurrenceID	An identifier for the Occurrence (as opposed to a particular digital record of the occurrence).
basisOfRecord	The specific nature of the data record.
eventDate	TThe date-time or interval during which an Event occurred. For occurrences, this is the Year-Month-Day format (YYYY-MM-DD) when the event was recorded.
year	The four-digit year in which the Event occurred, according to the Common Era Calendar.
month	The integer month in which the Event occurred.
day	The integer day of the month on which the Event occurred.
verbatimEventDate	The verbatim original representation of the date and time information for an Event.
eventRemarks	Comments or notes about the Event.
scientificName	An identifier for the nomenclatural (not taxonomic) details of a scientific name.
kingdom	The full scientific name of the kingdom in which the taxon is classified.
phylum	The full scientific name of the phylum or division in which the taxon is classified.
class	The full scientific name of the class (i.e. Insecta) in which the taxon is classified.
order	The full scientific name of the order in which the taxon is classified.
family	The full scientific name of the family in which the taxon is classified.
genus	The full scientific name of the genus in which the taxon is classified.
specificEpithet	The name of the first or species epithet of the scientificName.
taxonRank	The taxonomic rank of the most specific name in the scientificName.
identifiedBy	A list (concatenated and separated) of names of people, groups or organisations who assigned the Taxon to the subject.
institutionID	An identifier for the institution having custody of the object(s) or information referred to in the record i.e. http://grscicoll.org/institution/schmaulhausen-institute-zoology
continent	The name of the continent in which the Location occurs.
country	The name of the country or major administrative unit in which the Location occurs.
countryCode	The standard code for the country in which the Location occurs.
stateProvince	The name of the next smaller administrative region than country (state, province, canton, department, region etc.) in which the Location occurs.
county	The full, unabbreviated name of the next smaller administrative region than stateProvince (county, shire, department etc.) in which the Location occurs.
municipality	The full, unabbreviated name of the next smaller administrative region than county (city, municipality etc.) in which the Location occurs.
locality	The specific description of the place.
verbatimLocality	The original textual description of the place.
habitat	A category or description of the habitat in which the Event occurred.
decimalLatitude	The geographic latitude (in decimal degrees, using the spatial reference system given in geodeticDatum) of the geographic centre of a Location. Positive values are north of the Equator, negative values are south of it. Legal values lie between -90 and 90, inclusive.
decimalLongitude	The geographic longitude (in decimal degrees, using the spatial reference system given in geodeticDatum) of the geographic centre of a Location. Positive values are east of the Greenwich Meridian, negative values are west of it. Legal values lie between -180 and 180, inclusive.
geodeticDatum	The ellipsoid, geodetic datum or spatial reference system (SRS) upon which the geographic coordinates given in decimalLatitude and decimalLongitude are based.
coordinateUncertaintyInMeters	The horizontal distance (in metres) from the given decimalLatitude and decimalLongitude describing the smallest circle containing the whole of the Location.
verbatimCoordinates	The verbatim original spatial coordinates of the Location. The coordinate ellipsoid, geodeticDatum or full Spatial Reference System (SRS) for these coordinates should be stored in verbatimSRS and the coordinate system should be stored in verbatimCoordinateSystem.
verbatimCoordinateSystem	The coordinate format for the verbatimLatitude and verbatimLongitude or the verbatimCoordinates of the Location.
georeferencedBy	A list (concatenated and separated) of names of people, groups or organisations who determined the georeference (spatial representation) for the Location.
georeferencedDate	The date on which the Location was georeferenced.
georeferenceProtocol	A description or reference to the methods used to determine the spatial footprint, coordinates and uncertainties.
associatedReferences	A list (concatenated and separated) of identifiers (publication, bibliographic reference, global unique identifier, URI) of literature associated with the Occurrence.
organismQuantity	A number or enumeration value for the quantity of organisms.
organismQuantityType	The type of quantification system used for the quantity of organisms.
sex	The sex of the biological individual(s) represented in the Occurrence.
lifeStage	The age class or life stage of the Organism(s) at the time the Occurrence was recorded.
associatedTaxa	A list (concatenated and separated) of identifiers or names of taxa and the associations of this Occurrence to each of them.
establishmentMeans	Statement about whether an organism or organisms have been introduced to a given place and time through the direct or indirect activity of modern humans.
type	The nature or genre of the resource.
language	The language of the resource i.e. English.
license	A legal document giving official permission to do something with the resource.
institutionCode	The name (or acronym) in use by the institution having custody of the object(s) or information referred to in the record.
collectionCode	The name, acronym, coden or initialism identifying the collection or dataset from which the record was derived.
recordedBy	A list (concatenated and separated) of names of people, groups or organisations responsible for recording the original Occurrence.

## Additional information

The present article is the first attempt in biodata mobilisation of the entomological part of the SIZK Fund collections, which include millions of dry specimens and permanent slides. The presented material combines 11 datasets covering 11 insect families and 12,713 total occurrence records based on specimen label data.

### Sciaridae dataset

The Sciaridae dataset contains the biodata of specimens that are permanent micropreparations (slides) mounted and fixed in the artificial resin Euparal. Each slide contains only one male imago (adult) sciarid and only in very few cases the slides presented contain several imagines (males and/or females) or an additional empty puparium. The Sciaridae collection of SIZK covers almost the whole territory of Ukraine (except Crimea and Luhansk Region) and different types of habitats, both natural and anthropogenic (Babytskiy et al. 2019b, Babytskiy et al. 2023). Some specimens were collected in neighbouring countries, such as Hungary (Babytskiy et al. 2019a). The identification of all species in the dataset was verified by comparison with the voucher specimens and confirmed by the leading taxonomists of Sciaridae, Frank Menzel (Senckenberg Deutsches Entomologisches Institut, Müncheberg, Germany) and Pekka Vilkamaa (Finnish Museum of Natural History, Helsinki, Finland).

Successful identification to species level in Sciaridae is only possible based on careful study of the male imago, particularly the structure of the genitalia. Therefore, only males are useful for further preparation and examination (Babytskiy et al. 2022a).

Sciarids or black fungus gnats are small (imago length up to 8 mm), mainly dark-coloured insects. Their larvae usually develop in decaying plant remains permeated by fungal hyphae. Typical habitats of sciarids are shady forests and wet meadows, but some species can migrate from natural biotopes to anthropogenic ecosystems and live as synanthropes (Babytskiy and Bezsmertna 2021, Babytskiy et al. 2022b). Therefore, our Sciaridae collections focused on different grassland and forest habitats (Skobel et al. 2023).

The material was collected during various expeditions and excursions carried out in the time of the field seasons of 2012-2022 (Babytskiy et al. 2018, Babytskiy et al. 2020). Adult sciarids were collected using a sweepnet, Malaise and yellow pan traps or directly from the substrate using an aspirator. Collected sciarids were stored in 5 ml vials containing 70% ethanol. During laboratory preparation, male specimens were dehydrated in absolute ethanol and then mounted on slides in Euparal. All material is kept in the SIZK as a separate Sciaridae collection.

### Syrphidae dataset

The Syrphidae dataset contains the biodata of a collection of syrphids that are kept in the Funds of the I. I. Schmalhausen Institute of Zoology NAS of Ukraine (SIZK_Syrphidae). The collection consists of two parts: the first part contains slide mounted and pinned specimens; in the second part of the collection, there are only pinned specimens (without slide mounted material) and this is a comparative material necessary for identification of species. The terminalia of males are usually brought forward using a dissecting needle with a hook on the end to make the terminal sclerites easier to examine. In many cases, this makes it easier to identify questionable species. The Syrphidae collection of SIZK covers almost the whole territory of Ukraine (there are practically no collection data from the central regions of Ukraine) and different types of habitats, both natural and anthropogenic.

The material was collected during a series of expeditions and excursions carried out in the time of the field seasons from March to October (mostly April-August). The main biotopes in which Syrphidae were collected are edges of mixed or deciduous forests, floodplain forests or meadows, swamps, orchards and roadsides. The flies were mainly collected by the standard method using an entomological net or sometimes an aspirator. Species identification was carried out using the general identification keys published in the works of Bartsch et al. (2009a), Bartsch et al. (2009b), Van Veen 2010 and, Speight and Sarthou 2017, as well as identification keys for individual genera in the works of various specialists. All material is kept in the SIZK Funds as a separate Syrphidae collection.

### Tortricidae dataset

The Tortricidae dataset contains the biodata of tortrix moth specimens, which are mounted with entomological pins and minutiae. Prepared chitinised genital structures of males and females are stored in special plastic tubes in a mixture of alcohol and glycerine. The SIZK Tortricidae collection covers the territory of sixteen regions of Ukraine (Cherkasy, Chernihiv, Dnipropetrovsk, Donetsk, Kharkiv, Kherson, Kyiv, Luhansk, Lviv, Mykolaiv, Odesa, Sumy, Volyn, Zakarpatska, Zaporizhzhya and Zhytomyr Regions) and the Autonomous Republic of Crimea. Some specimens were collected in countries of the former Soviet Union, such as Azerbaijan, Belarus, Kazakhstan, Kyrgyzstan and the Russian Federation. The specimens presented in the collection were collected between 1916 and 2016 in various habitat types both natural and anthropogenic. The identification of all species in the dataset has been verified by comparison with voucher specimens and confirmed by leading taxonomists of Tortricidae.

The Tortricidae are a family of moths, commonly known as tortrix moths or leafroller moths, in the order Lepidoptera. This large family has over 11,000 described species and is the only member of the superfamily Tortricoidea. Many species are economically important pests. The typical resting posture is with the wings folded back, producing a rather rounded profile.

Larvae in the subfamilies Chlidanotinae and Olethreutinae usually feed by boring into stems, roots, buds or seeds. Larvae in the subfamily Tortricinae feed externally, building leaf rolls. Larvae in the subfamily Tortricinae tend to be more polyphagous than those in Chlidanotinae and Olethreutinae. Tortricinae also possess an anal fork to flick excrement away from their shelter.

The material was collected in a number of expeditions and excursions carried out during the field seasons of the years 1916–2016 in various habitat types both natural and anthropogenic. Adult tortrix moths were observed and collected via daytime catching by a net, attracted by light (250 W and 500 W mercury arc lamps, in rare cases, also incandescent lamps). The caught specimens were placed in individual test tubes, which were closed with a cotton swab and euthanised with ethyl acetate. After the excursions, the best collected specimens were pricked on entomological pins (No. 000 and No. 00) or on minutiae and part of the material was laid out on cotton layers ('mattresses'). When attracted by light, tortrix moths that landed on a white screen were collected in test tubes, euthanised and mounted immediately after catching. When inspecting tree trunks, walls of houses, fences etc., tortrix moths sitting on them were caught with test tubes or stains.

The identification of the material was carried out mainly using the literature sources (Kuznetzov 1978, Razowski 2002, Schön 2002, Razowski 2003), and was verified by comparison with the voucher specimens from the Yu. Kostiuk collection of tortrix moths of the National Museum of Natural History at the National Academy of Sciences of Ukraine (Kyiv). In difficult cases, the identification was made by genitalia prepared by maceration in 8–12% sodium hydroxide (NaOH) or potassium hydroxide (KOH) solution. Prepared chitinised genital structures of males and females are stored in special plastic tubes in a mixture of alcohol and glycerine. All of the material is kept in the SIZK Funds as a separate Tortricidae collection.

### Chrysididae dataset

The Chrysididae dataset contains the biodata on the collection of the family Chrysididae kept in the Funds of I.I. Schmalhausen Institute of Zoology NAS of Ukraine (SIZK_Chrysididae) and partially originating from Kharkiv Entomological Society (Kharkiv, Ukraine), Nature Museum of V.N. Karazin Kharkiv National University (Kharkiv, Ukraine), Vasyl` Stus Donetsk National University (Donetsk, Ukraine) and Karadag Nature Reserve (The Autonomous Republic of Crimea, Ukraine)). The dataset comprises data on specimens collected since 1873 to now. Chrysidid wasps were mainly studied as imagoes, but some data refer to larval stage and subsequently reared adults. The vast part of studied specimens is preserved as pinned material, some of them also being preserved in 96% ethanol. In a geographical aspect, the collection covers almost the entire territory of Ukraine: it represents data from all geographic and administrative regions of the country. Identification of many species in the dataset was verified by comparison with the voucher specimens and confirmed by the leading taxonomists on Chrysididae.

The minute study of an adult is usually sufficient for the successful identification of the species of the family Chrysididae. The known ranges of variability in colouration and morphological features should be accurately considered in the process of differentiating (Martynova 2021). Nevertheless, the structure of male genitalia and female internal segments are used for identification of the species of certain species-groups within Chrysididae. Various recent keys were used for the identification of species of different genera and species-groups of the family (Kimsey and Bohart 1991, Smissen 2010, Martynova 2015).

The material on Chrysididae was collected during numerous expeditions all over Ukraine. Different entomological methods were used for capturing of the adults, for example, sweep-netting, collecting of separate individuals of flowers, wood, leaves and other surfaces, yellow pan traps, Malaise traps and Barber traps. Additionally, rearing of adults from the host nests was used in the course of study on host-parasitoid relations of Chrysidid wasps (Martynova 2021).

The captured specimens were narcotised in the field with ethylene glycol or preserved directly in 96% ethanol. Afterwards, they were pinned in the standard way at laboratory conditions for further examination. The material is kept in the SIZK Funds as a separate Chrysididae collection. It also comprises specimens originating from collections of other institutions (Nature Museum of V.N. Karazin Kharkiv National University (Kharkiv, Ukraine), Vasyl` Stus Donetsk National University (Donetsk, Ukraine) and Karadag Nature Reserve (The Autonomous Republic of Crimea, Ukraine)).

### Larrini and Miscophini dataset

The Larrini and Miscophini dataset contains the biodata of the specimens belonging to these tribes (Crabronidae family). All specimens are pinned, fixed and dried. Each specimen is accompanied by labels. Tribes Larrini and Miscophini have been collected from parts of Ukraine territory (Autonomous Republic of Crimea, Chernihiv, Donetsk, Kherson, Kyiv, Luhansk, Mykolaiv, Odesa, Poltava, Volyn, Zaporizhzhia and Zhytomyr Regions) and various habitat types both natural and anthropogenic. Some specimens have also been collected in other countries such as Mongolia, Kazakhstan, Kyrgyzstan, Tajikistan, Turkmenistan, Armenia, Azerbaijan, Moldova and the Russian Federation.

Successful identification to the species level in Larrini and Miscophini is possible only based on carefully studying the morphology(Bohart and Menke 1976, Kazenas 1978, Pulawski 1978).

Larrini and Miscophini live in a wide range of habitats, both natural and anthropogenic (Kazenas 1978). Biology of species from these tribes is presented in detail in the monograph of V. Kazenas (Kazenas 2001).

The material was collected in a number of expeditions and excursions carried out during the field seasons of the years 1912 – 1992. Adult Tribes Larrini and Miscophini were collected with a sweepnet and Malaise traps. Collected insects were stored in vials with ethyl acetate vapour. During preparation in the laboratory, the specimens were pinned, fixed, dried and labelled. All of the material is kept in the SIZK Funds as a part of Crabronidae collection.

### Crabronidae and Sphecidae dataset

The Crabronidae and Sphecidae dataset contains the biodata of specimens from two Hymenoptera families. The family Crabronidae (864 specimens) is represented by 99 species and two subspecies from 18 genera. The Sphecidae family (206 specimens) is represented by 23 species from six genera. All specimens are pinned, fixed and dried. Each specimen is accompanied by labels. Crabronidae and Sphecidae SIZK collections cover almost the whole territory of Ukraine and the territory of the CIS countries (Republics of the former USSR). Сollections cover habitat types both natural and anthropogenic.

Biotopes for most specimens are not specified. For some specimens, the following biotopes were indicated: steppe, forest-steppe, mountains, sands, gardens, forest, forest edges, river and lake valleys, sea coasts, xerophilic meadows, ravines and anthropogenic landscapes (Bohart and Menke 1976, Kazenas 1978, Kazenas 2001). The material was collected in a number of expeditions and excursions carried out during the field seasons of the years 1905 – 2007. During preparation in the laboratory, the collected insects were pinned, fixed, dried and labelled. All of the material is kept in the SIZK Funds as separate Crabronidae and Sphecidae collections.

### Eurytomidae, Torymidae and Ormyridae dataset

The Eurytomidae, Torymidae and Ormyridae dataset contains the biodata of the superfamily Chalcidoidea (Hymenoptera, Eurytomidae, Torymidae, Ormyridae) collections. All specimens in the dataset are pinned imagoes (adults). Collections of Eurytomidae, Torymidae and Ormyridae cover the territory of western Ukraine (Carpathian Mountains), northern and central Ukraine, eastern and southern Ukraine), including various natural reservations in forestry, forest-steppe and steppe natural regions of Ukraine (Zerova 1995).

Identification of all species in the dataset was verified by comparison with the voucher specimens (both type and non-type) and confirmed by the leading taxonomists on different families of Chalcidoidea (Zerova and Klymenko 2018).

Chalcid wasps (Chalcidoidea) have a small body size (mostly length of imago from 0.3 mm to 6.0 mm without ovipositor length). Their larvae usually develop as ecto- or endoparasitoids, koino- or idiobionts, gregarious or solitary parasitoids and, in some cases, larvae are phytophagous in seeds, fruits, stems, leaves and galls of different plants. Chalcid wasps can be found in all terrestrial habitat types, including forests, shrubs, forestry-steppe and steppe landscapes, as well as near semi-aquatic and even aquatic habitats (Bolu et al. 2024).

The material was collected during 1899 – 2017 by many entomologists. Adults of chalcid wasps were collected with a sweepnet, Malaise and yellow pan traps. Collected in traps, parasitoids are stored in 2.0-50.0 ml vials containing 96% ethanol. During preparation in the laboratory, the specimens were dried and then pinned directly or glued to paper triangles with further pinning. Extensive material of chalcid wasps was received by the method of individual rearing from host insects and host plants. All of the material is kept in the SIZK Funds as separate Eurytomidae, Torymidae and Ormyridae collections containing the specimens collected in all parts of the world.

### Ichneumonidae dataset

The Ichneumonidae dataset contains the biodata of specimens from Ichneumonidae family. All specimens are pinned or preserved in ethanol imagoes (adult). Ichneumonidae collection of SIZK covers the territory of western Ukraine (part of the Carpathian Mountains situated in Ivano-Frankivsk and Transcarpathian Regions) and various natural forest habitat types. Identification of all species in the dataset was verified by comparison with the voucher specimens (both type and non-type) and confirmed by the leading taxonomists of Ichneumonidae (e.g. Varga (2024)).

Successful identification to the species level in Ichneumonidae is usually possible based on careful studying of the pinned female imago. In some well-studied groups, such as subfamily Pimplinae, identification is possible based on wet (preserved in ethanol) specimens of both sexes without preliminary pinning (Varga 2019). Thus, a large part of material is stored in vials filled with ethanol.

Ichneumonids or Darwin wasps are small to large (length of imago from 2 mm to 50 mm without ovipositor lenght). Their larvae usually act as parasitoids developing as ecto- or endoparasitoids, koino- or idiobionts, gregarious or solitary. Ichneumonids can be found in all terrestrial habitat types, but they are the most abundant in wet forests (Broad et al. 2018).

The material was collected in a number of expeditions carried out during the field seasons of the years 2009 – 2018. Adult ichneumonids were collected with a sweepnet, Malaise and Trunk traps. Collected parasitoids were stored in 2-50 ml vials containing 96% ethanol. During preparation in the laboratory, the specimens were dried and then pinned directly or glued to paper triangles with further pinning. All of the material is kept in the SIZK Funds as a part of Ichneumonidae collection containing the specimens collected in all parts of the world.

### Anomaloninae dataset

The Anomaloninae dataset contains the biodata of Anomaloninae subfamily (Hymenoptera, Ichneumonidae). All specimens are pinned, fixed and dried. Each specimen is accompanied by labels. Anomaloninae SIZK collection covers almost the whole territory of Ukraine and various habitat types both natural and anthropogenic (Nuzhna 2010a, Nuzhna 2010b, Nuzhna 2012a, Nuzhna 2012b, Nuzhna 2013a, Nuzhna 2013b, Nuzhna 2013c). Specimens were collected in neighbouring countries, such as Belarus, Russian Federation, Moldova and some specimens were collected in Kazakhstan (Nuzhna 2013c). Identification of all species in the dataset was verified by comparison with the voucher specimens and confirmed by the leading taxonomists of Anomaloninae.

Successful identification to the species level in Anomaloninae is possible only based on careful examination of the morphological characters, such as body sculpture, wing venation, shape of head, metasoma and hind tarsi. Colouration is also very useful especially for male determination.

Anomalonines are small to large, but always slender insects, with strongly sculptured propodeum and laterally compressed metasoma. The representatives of this group are koinobiont endoparasitoids of Coleoptera (Anomalonini) and Lepidoptera (Gravenhorstiini) (Broad et al. 2018). Typical habitats for Anomaloninae are dry or wet grassland and shrub biotopes, but some species can migrate from the natural biotopes to anthropogenic ecosystems and live as synanthropes. Therefore, our Anomaloninae collections focused on different grasslands and forest habitats (Nuzhna 2010b, Nuzhna 2013b).

The material was collected during the numerous field trips and excursions carried out by many entomologists between 1952 and 2012. Adult Anomaloninae were collected with a sweepnet and Malaise traps. Collected insects were stored in vials with ethyl acetate vapour. The specimens prepared to be stored in the main Ichneumonidae collection at SIZK were preliminary pinned, dried and labelled (Nuzhna 2010a, Nuzhna 2010b, Nuzhna 2012a, Nuzhna 2012b, Nuzhna 2013a, Nuzhna 2013b, Nuzhna 2013c).

### Ophioninae dataset

The Ophioninae dataset contains the biodata of Ophioninae subfamily (Hymenoptera, Ichneumonidae). All specimens are pinned, fixed and dried. Each specimen is accompanied by labels. Ophioninae SIZK collection covers almost the whole territory of Ukraine and various habitat types both natural and anthropogenic. Specimens were collected in neighbouring countries, such as the Russian Federation, Belarus, Moldova, Hungary and some specimens were collected in Indonesia, Peru, Madagascar.

The number of flagellomeres, shape of ocelli, wing venation and carination of the propodeum are the main features used for successful species identification of the Ophioninae (e.g. Johansson and Cederberg (2019)).

These insects are large and slender nocturnal parasitoids, usually with uniformly coloured (mainly orange in temperate zone species) body and long antennae. They act as koinobiont endoparasitoids of various lepidopterans (Broad et al. 2018). Typical habitats for Ophioninae are dry or wet grassland and shrub biotopes, but some species can migrate from the natural biotopes to anthropogenic ecosystems and live as synanthropes. Therefore, our Ophioninae collections focused on different grasslands and forest habitats.

The material was collected mainly with light traps, but sweepnet and Malaise traps were also used. The materials were represented by the specimens collected in 1907 – 2007. The material is kept as pinned specimens in the SIZK Funds as a part of the main Ichneumonidae collection.

### Aphidiinae dataset

The Aphidiinae dataset contains the biodata of the Aphidiinae subfamily collection (Hymenoptera, Braconidae). All specimens are fixed, dried and pinned (card mounting method). Each specimen is accompanied by label(s). Aphidiinae collection of SIZK covers most of the territory of Ukraine (18 regions) and various habitat types both natural and anthropogenic. Species were identified using classical and modern identification keys of the group (Tobias and Chiriac 1986, Davidian 2007) as well as keys to particular genera and original species descriptions, information on which could be found in Taxapad (Yu et al. 2016). Identification of some specimens was verified by comparison with the voucher specimens and confirmed by the leading taxonomists of Aphidiinae (Kaliuzhna 2014).

Successful identification to species level in Aphidiinae is only possible based on careful study of morphology, especially head, meso- and metasoma structure (Kaliuzhna 2014, Kaliuzhna 2016). Mostly females are useful for identification, but sometimes males could also be identified. In some cases, mounting on slides is necessary for species identification. Species identification, based on morphological characters in some cases, is difficult and often the use of DNA analysis provides new results on the species diversity (Havelka et al. 2021). The need to find new reliable characters has led to attempts to describe new morphological characters in Aphidiinae (Kaliuzhna 2016, Kaliuzhna 2019b).

Aphidiinae are slender, medium-sized to small insects (typically 2–4 mm). Imago colouration is predominantly dark brown, brown, yellow-brown and rarely yellow. Venation is often reduced compared to other braconids, except for the genera *Ephedrus* and *Toxares*. Ovipositor sheaths are always short. Antennae are usually not very long, often around or less than 20 flagelomeres. Aphidiines are koinobiont endoparasitoids of aphids (Hemiptera, Aphididae); some species are used as biological control agents (Starý 1970, Tobias and Chiriac 1986, Davidian 2007).

Typical habitats for Aphidiinae are dry or wet grasslands, ecotones between forest and meadow, field edges and ruderal habitats (Kaliuzhna and Zubenko 2013), but some species are parasitoids of dendrophilous aphids and could be found in forests or parks, arboretums or botanical gardens (Kaliuzhna 2012) and other species are associated with ants and could be found in their nests. Therefore, the collection of Aphidiinae was focused on different grassland and forest habitats, as well as agricultural and ruderal areas.

The material was collected in a number of expeditions and excursions during the field seasons of 1972 – 2019 (Kaliuzhna 2012, Kaliuzhna 2013, Kaliuzhna and Zubenko 2013, Kaliuzhna 2014, Kaliuzhna 2017, Kaliuzhna 2019a, Kaliuzhna 2019b, Kaliuzhna 2020). Adult Aphidiinae were collected with a sweepnet or reared from infested aphid hosts. Collected insects were fixed in vials with ethyl acetate vapour. During preparation in the laboratory, specimens were mounted on cards and labelled. All the material is kept in the SIZK Funds as a part of Braconidae collection.

## Supplementary Material

C77F5851-060B-5DAE-BC5D-F5A03B0EF5FD 10.3897/BDJ.13.e152004.suppl1 Supplementary material 1 The number of occurrences, species and specimens of certain families in Ukraine and other countries of the world presented in this article. Data type Table Brief description The number of occurrences, species and specimens of certain families in Ukraine and other countries of the world presented in this article. File: oo_1322421.xlsx https://binary.pensoft.net/file/1322421 Babytskiy A, Kaliuzhna M, Varga O, Kavurka V, Prokhorov A, Kletionkin V, Martynova K, Nuzhna A, Klymenko S, Fursov V, Parkhomenko M

## Figures and Tables

**Table 1.** The number of records of specific insect families in individual regions of Ukraine presented in this article.

Region of Ukraine	Total occurrences	Braconidae	Chrysididae	Crabronidae	Eurytomidae	Ichneumonidae	Ormyridae	Sciaridae	Sphecidae	Syrphidae	Tortricidae	Torymidae	Total check
Cherkasy	326	184	4	7	15	5	0	18	4	3	82	4	326
Chernihiv	897	1	23	43	3	34	0	3	4	104	679	3	897
Chernivtsi	95	4	7	0	9	0	0	66	0	7	0	2	95
Crimea	497	2	84	181	28	102	3	0	53	5	13	26	497
Dnipropetrovks	53	0	35	1	3	8	0	0	0	0	4	2	53
Donetsk	1709	1	1554	11	28	93	2	0	0	4	2	14	1709
Ivano-Frankivsk	192	0	22	7	6	119	0	13	0	24	0	1	192
Kharkiv	580	5	283	4	2	30	0	2	1	1	243	9	580
Kherson	376	18	24	40	26	214	1	0	7	31	5	10	376
Khmelnytskyi	45	0	5	0	5	15	2	1	0	13	0	4	45
Kirovohrad	71	32	12	6	2	5	0	0	14	0	0	0	71
Kyiv	3000	123	130	179	73	107	4	321	67	1627	314	55	3000
Luhansk	393	1	128	5	2	70	0	0	1	0	184	2	393
Lviv	43	0	13	0	6	5	0	13	0	0	1	5	43
Mykolaiv	108	6	20	9	4	61	0	0	2	1	4	1	108
Odesa	185	4	22	33	17	42	0	7	1	20	31	8	185
Poltava	75	34	14	5	0	14	0	1	6	0	0	1	75
Rivne	523	0	3	0	0	7	0	3	0	509	0	1	523
Sumy	306	8	12	1	3	6	0	0	0	64	211	1	306
Ternopil	340	23	26	3	1	1	0	273	0	11	0	2	340
Vinnytsia	70	0	4	1	3	8	0	34	12	3	0	5	70
Volyn	298	1	26	6	5	6	0	207	1	43	2	1	298
Zakarpatska	1198	1	4	12	10	917	3	28	0	184	6	33	1198
Zaporizhzhia	209	1	8	5	4	49	0	0	0	2	136	4	209
Zhytomyr	163	0	25	10	1	3	0	0	0	117	7	0	163
Total	11752	449	2488	569	256	1921	15	990	173	2773	1924	194	11752
